# Bile Acids Gate Dopamine Transporter Mediated Currents

**DOI:** 10.3389/fchem.2021.753990

**Published:** 2021-12-10

**Authors:** Tiziana Romanazzi, Daniele Zanella, Mary Hongying Cheng, Behrgen Smith, Angela M. Carter, Aurelio Galli, Ivet Bahar, Elena Bossi

**Affiliations:** ^1^ Department of Biotechnology and Life Sciences, University of Insubria, Varese, Italy; ^2^ Department of Surgery, University of Alabama at Birmingham, Birmingham, AL, United States; ^3^ Department of Computational and Systems Biology, School of Medicine, University of Pittsburgh, Pittsburgh, PA, United States; ^4^ Department of Physics and Chemistry, Biomolecular Engineering, Milwaukee School of Engineering, Milwaukee, WI, United States; ^5^ Center for Research in Neuroscience, University of Insubria, Varese, Italy

**Keywords:** bile acid, dopamine, monoamines, transporters, SLC6, electrophysiology

## Abstract

Bile acids (BAs) are molecules derived from cholesterol that are involved in dietary fat absorption. New evidence supports an additional role for BAs as regulators of brain function. Sterols such as cholesterol interact with monoamine transporters, including the dopamine (DA) transporter (DAT) which plays a key role in DA neurotransmission and reward. This study explores the interactions of the BA, obeticholic acid (OCA), with DAT and characterizes the regulation of DAT activity *via* both electrophysiology and molecular modeling. We expressed murine DAT (mDAT) in *Xenopus laevis* oocytes and confirmed its functionality. Next, we showed that OCA promotes a DAT-mediated inward current that is Na^+^-dependent and not regulated by intracellular calcium. The current induced by OCA was transient in nature, returning to baseline in the continued presence of the BA. OCA also transiently blocked the DAT-mediated Li^+^-leak current, a feature that parallels DA action and indicates direct binding to the transporter in the absence of Na^+^. Interestingly, OCA did not alter DA affinity nor the ability of DA to promote a DAT-mediated inward current, suggesting that the interaction of OCA with the transporter is non-competitive, regarding DA. Docking simulations performed for investigating the molecular mechanism of OCA action on DAT activity revealed two potential binding sites. First, in the absence of DA, OCA binds DAT through interactions with D421, a residue normally involved in coordinating the binding of the Na^+^ ion to the Na2 binding site (Borre et al., J. Biol. Chem., 2014, 289, 25764–25773; Cheng and Bahar, Structure, 2015, 23, 2171–2181). Furthermore, we uncover a separate binding site for OCA on DAT, of equal potential functional impact, that is coordinated by the DAT residues R445 and D436. Binding to that site may stabilize the inward-facing (IF) open state by preventing the re-formation of the IF-gating salt bridges, R60-D436 and R445-E428, that are required for DA transport. This study suggests that BAs may represent novel pharmacological tools to regulate DAT function, and possibly, associated behaviors.

## Introduction

Bile acids (BAs) are amphipathic molecules derived from cholesterol that are primarily synthesized in the liver and stored in the gallbladder. Upon food consumption and transit, BAs are released into the duodenum (Mertens et al., 2017) where their main physiological role is solubilization and absorption of dietary fat. Administration of BAs has been developed into successful therapies for the treatment of liver and gallbladder pathologies, such as non-alcoholic steatohepatitis and cholelithiasis (Cruz-Ramon et al., 2017; Li and Chiang, 2020). When administered orally, they exhibit favorable bioavailability. They are readily absorbed through the portal vasculature and distributed throughout the body (Kiriyama and Nochi, 2019; Fiorucci et al., 2021).

Receptors for BAs are present throughout the brain (Maruyama et al., 2002; Kawamata et al., 2003; Keitel et al., 2010; Huang et al., 2015; Perino et al., 2021) and evidence exists for their synthesis directly in the CNS (McMillin and DeMorrow, 2016; Mertens et al., 2017; Kiriyama and Nochi, 2019). This raises the possibility of physiologic roles for BAs other than acting as an adjuvant in fat absorption, such as regulators of CNS activity. Consistent with this idea, freely circulating BAs have been implicated in the modulation of CNS proteins such as NMDA, GABA_A_ and M_3_ muscarinic receptors (Raufman et al., 2002; Schubring et al., 2012), the activation of neuronal ion channels (Wiemuth et al., 2014; Kiriyama and Nochi, 2019) and stimulation of the release of neuroactive peptides such as GLP-1 (Flynn et al., 2019; Chaudhari et al., 2021; Fiorucci et al., 2021). BAs pass the blood-brain barrier (BBB) through passive diffusion, as well as through active membrane transporters, and BA levels in the brain have been correlated to plasma levels (Mano et al., 2004; Higashi et al., 2017; Reddy et al., 2018; Kiriyama and Nochi, 2019). The number of studies proposing BAs as a treatment for brain disorders are steadily increasing (Bhargava et al., 2020; Wu et al., 2020; Huang et al., 2021; Jin et al., 2021; Zangerolamo et al., 2021) but understanding of their mechanisms of action is still limited.

BA concentrations vary in the body following a circadian rhythm and are highly influenced by the fasting vs fed state (Fiorucci et al., 2021; Perino et al., 2021). Changes in circulating BAs are also promoted by pathological alterations in liver functionality and metabolism (Fiorucci et al., 2021). Multiple types of bariatric surgery increase circulating BAs and, in mice, cause loss of preference for dietary fat (Scholtz et al., 2014; Bensalem et al., 2020) and, interestingly, cocaine (Reddy et al., 2018). Further, feeding BAs to mice recapitulated the effects of bariatric surgery on cocaine preference, strongly supporting their ability to modulate reward circuitries in the CNS as well as dopamine (DA) neurotransmission (Reddy et al., 2018). Understanding the molecular determinants of these actions could facilitate BA-based therapies for drug use disorders as well as identify potential new therapeutic targets.

BAs are directly derived from cholesterol and retain a high degree of structural similarity with the parent molecule but differ by the presence of various R groups, many of which are charged, added to the A, B, and C rings as well as the hydrocarbon tail. Resulting BAs are less lipophilic and more soluble in aqueous solutions relative to cholesterol (Russell, 2003). The importance of cholesterol in monoamine transporter (MAT) function and membrane localization is well documented (Foster et al., 2008; Chang and Rosenthal, 2012). Cholesterol depletion modulates both MAT expression (Butchbach et al., 2004; Magnani et al., 2004; Foster et al., 2008) and activity (Magnani et al., 2004; Adkins et al., 2007; Foster et al., 2008; Hong and Amara, 2010). There are indications of direct interactions between cholesterol and MATs such as the DA transporter (DAT) and human serotonin transporter (hSERT) (Cremona et al., 2011; Jones et al., 2012; Penmatsa et al., 2013; Wang et al., 2015; Coleman et al., 2016; Zeppelin et al., 2018) whereas no information is available on interactions occurring between BAs and MATs. Thus, investigations of the functional relevance of interactions between cholesterol-like molecules and MATs are needed to further define the central regulatory role of these sterols. This study demonstrates the ability of BAs to alter DAT activity and identifies putative binding sites that may underlie the structural changes driving these alterations.

## Materials and Methods

### Solutions

Composition of buffered solution (ND96) (in mM): NaCl 96, KCl 2, CaCl_2_ 1.8, MgCl_2_ 1, HEPES 5, pH 7.6. Composition of NDE solution: ND96 plus 2.5 mM pyruvate and 50 μg/ml Gentamycin sulphate. Composition of external control buffer (ND98) (in mM): NaCl 98, MgCl_2_ 1, and CaCl_2_ 1.8 with or without 0.01% DMSO. In tetramethylammonium (TMA)-chloride “zero sodium” buffer (TMA98), equimolar TMACl replaces NaCl. In Li^+^ buffer, equimolar LiCl replaces NaCl. The final pH was adjusted using respective hydroxides (NaOH or TMAOH or LiOH) to 7.6 for all external solutions. Substrates used were Dopamine (DA) (Calbiochem - Sigma, Milan, Italy), Lithocholic acid (LCA) (Sigma), and Obeticholic acid (OCA) (Adipogen, Switzerland). LCA or OCA powder was dissolved in DMSO at 50 mM and 100 mM, respectively.

### Oocytes Collection and Preparation

Oocytes were obtained from adult *Xenopus laevis* females. Animals were anaesthetised in 0.1% (w/v) MS222 (tricaine methanesulfonate; Sigma) solution in tap water. Abdomens were sterilized with antiseptic agent (Povidone-iodine 0.8%), laparotomy was performed, and portions of the ovary were collected. The oocytes were treated with 0.5 mg/ml collagenase (Sigma Type IA) in ND96 calcium-free for at least 30 min at 18°C. Healthy and fully-grown oocytes were selected and stored at 18°C in NDE solution (Bossi et al., 2007). The day after the removal, the oocytes were injected with cRNA using a manual microinjection system (Drummond Scientific Company, Broomall, PA). Injected concentrations were 12.5 ng/50 nl for the mouse dopamine transporter (mDAT), 2 ng/50 nl for human Takeda G protein-coupled receptor (hTGR5).

### cRNA Preparation

mDAT cDNA in pcDNA3.1 was kindly gifted from Prof. Dr. Harald Sitte of Medical University of Vienna. The cDNA mDAT was amplified with forward and reverse primers containing SmaI and EcoRI restriction sites, respectively (5′-GAC​TCCC​GGGACC​CAT​GAG​TAA​AAG​CAA​ATG-3′; 5′-GCA​TGAA​TTCTTA​CAG​CAA​CAG​CCA​ATG​GCG​C-3′). The amplified coding sequence was then subcloned into the pGHJ vector after double digestion with SmaI e EcoRI restriction enzymes (Promega). hTGR5 gene was in pCMV6-Entry (GPBAR1 Human cDNA ORF Clone, NM_001077191; Origene Technologies, Inc., Rockville, MD, United States). The two plasmids were linearized with SalI (mDAT) and with NdeI (hTGR5), *in vitro* capped, and transcribed using T7 RNA polymerase. Enzymes were supplied by Promega Italia. The oocytes were incubated at 18°C for 2–3 days prior to electrophysiological experiments. The experimental protocol was approved locally by the Committee of the “Organismo Preposto al Benessere degli Animali” of the University of Insubria (OPBA-permit #02_15) and nationally by Ministero della Salute (permit nr. 1011/2015).

### Electrophysiology

Electrophysiological studies were performed using the Two-Electrode Voltage Clamp (TEVC) technique (Oocyte Clamp OC-725; Warner Instruments, Hamden, CT, United States). Controlling software was WinWCP version 4.4.6 (J. Dempster, University of Strathclyde, United Kingdom) or Clampex (Molecular Devices, Sunnyvale, CA, United States, www.moleculardevices.com). Borosilicate microelectrodes, with a tip resistance of 0.5–4 MΩ, were filled with 3 M KCl. Bath electrodes were connected to the experimental oocyte chamber via agar bridges (3% agar in 3 M KCl). The holding potential was kept at −60 mV for all the experiments. The minimal concentration of OCA inducing inward current was evaluated by applying concentration of OCA from 1 pM to 100 µM. Each dose was tested on one single oocyte, exposed before to 30 µM DA. The current in the presence of OCA is the maximal current measured at the peak. For the I/V relationships, a step protocol from −160 mV to +40 mV with 20 mV of increment was applied for 100 ms. The mean of the transport-associated currents plotted in the scatter diagrams and in the I/V relationships were determined by subtracting the current recorded in the ND98 buffer from the current recorded in the presence of DA or OCA. In experiments using TMA, subtraction was performed with the current in TMA98 buffer. To chelate intracellular calcium, oocytes were injected with 50 nl of an intracellular solution containing 13 mM EGTA 30 min prior to electrophysiological recording. Intracellular solution had the following composition (in mM): KCl 130, NaCl 4, MgCl_2_ 1.6, HEPES 10, glucose 5, pH 7.6. The dose-response experiments in Figure 4 were performed exposing the same oocytes first to all the concentrations of DA alone, then to the same concentrations plus 10 µM OCA. Data analysis was performed using Clampfit 10.2 software (Molecular Devices, Sunnyvale, CA, United States, www.moleculardevices.com); OriginPro 8.0 (OriginLab Corp., Northampton, MA, United States, www.originlab.com) and GraphPad Prism (www.graphpad.com/scientific-software/prism) were used for statistical analysis and figure preparation.

### Structural Models for mDAT and hDAT

The mDAT (residues R56 to N596; UniProt ID O55192) in the outward-facing open (OF*o*), occluded, and inward-facing open (IF*o*) states were generated using SWISS-MODEL (Waterhouse et al., 2018) based on the structures resolved for OF*o* Drosophila DAT (dDAT) (PDB: 4M48) (Penmatsa et al., 2013), occluded hSERT (PDB: 6DZV) and IF*o* hSERT (PDB: 6DZZ) (Coleman et al., 2019). Homology models for hDAT in these conformational states were taken from previous work (Cheng et al., 2018; Aggarwal et al., 2021).

### Docking Simulations

OCA 3D structures were downloaded from the ZINC database (Irwin and Shoichet, 2005) (ZINC14164617) and DrugBank (Wishart et al., 2006) (DB05990). The net charge of OCA is indicated to be −1 in the ZINC database, and zero in DrugBank. Docking simulations were performed using both electronic states, designated as OCA(−) and OCA(n) for the negatively-charged and neutral OCAs, respectively. The binding sites and binding poses on both mDAT and hDAT, in different conformational states, were assessed using docking simulation software AutoDock4 (Morris et al., 2009) and AutoDock Vina (Trott and Olson, 2010). Autodock Vina simulations were carried out using a grid with dimensions set to 84 × 58 × 86 Å^3^ and center at (−1.22, 1.16, −6.26 Å) for each conformer and each transporter. This grid box encapsulated the entire structure of the transporter. The number of runs (exhaustiveness parameter in Autodock Vina) was set to 50 and the algorithm returned 20 binding modes of interest for each conformer. AutoDock4 simulations were performed following previously published protocols (Cheng et al., 2015; Aggarwal et al., 2021). Briefly, Lamarckian genetic algorithm with default parameters was employed with the maximal number of energy evaluations set to 2.5 × 10^7^. The binding energy was estimated from the weighted average of multiple binding poses at a given site observed in 100 independent runs.

## Results

### OCA Induces a DAT-Dependent, Transient Inward Current

**FIGURE 1:**
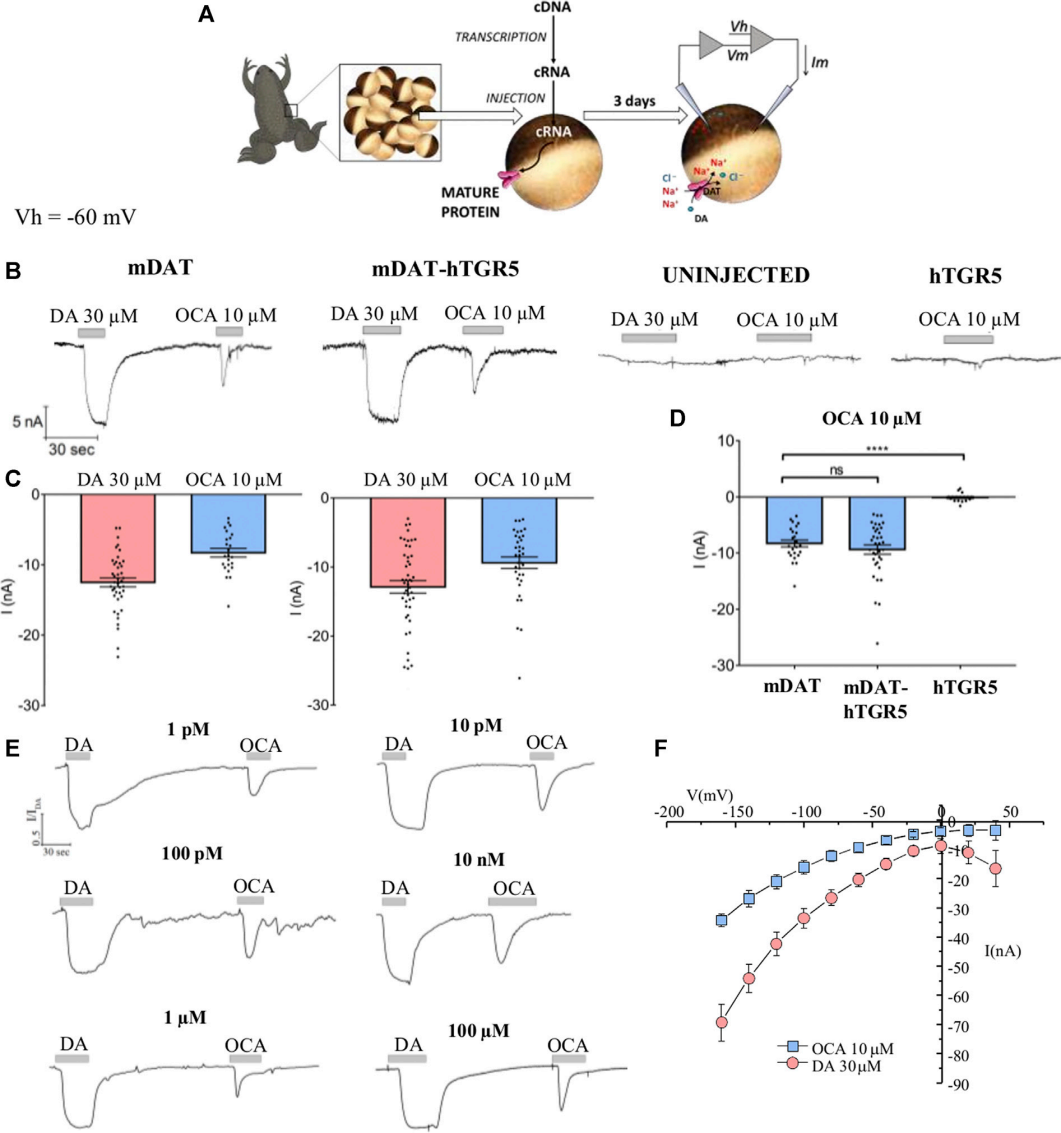
OCA generates an electrical current through the DAT. **(A)** Schematic representation of oocyte collection, cRNA synthesis and injection, and TEVC technique. **(B)** Representative traces of currents recorded by TEVC (Vh = −60 mV) from oocytes expressing mDAT, without or with hTGR5, uninjected, or expressing hTGR5 alone. The oocytes were perfused with 30 µM DA or 10 µM OCA. **(C)** Mean of the maximal DA-associated and OCA-induced currents (I nA ± SE of 14–47 oocytes, 6–11 batches) in oocytes expressing mDAT **(C, left)** or mDAT plus TGR5 **(C, right)**. **(D)** Mean of the maximal OCA-induced currents in oocytes expressing the proteins indicated; one-way ANOVA F(2,74) = 36.13, *p* < 0.0001 followed by Tukey’s multiple comparison test (mDAT vs mDAT-hTGR5 *p* = 0.566, mDAT vs hTGR5 *p* < 0.0001). **(E)** Representative traces of currents recorded by TEVC (Vh = −60 mV) from oocytes tested with DA (30 µM) and OCA at the indicated concentrations. The current of each trace was normalized to the mean current of dopamine to reduce the variability between batches. **(F)** I/V relationships of DA (30 µM) and OCA (10 µM)-induced currents.

To begin to investigate the regulatory effects of OCA on DAT electrical activity, *Xenopus laevis* oocytes were tested by two-electrode voltage clamp (TEVC) (Figure 1A). Perfusion of DA onto the oocytes expressing the mouse DAT (mDAT) generated an inward current (−12.5 nA ± 0.63) (Figures 1B,C, left), confirming the expression and functionality of the transporter. On the same oocytes, following DA washout, perfusion of 10 µM OCA also generated an inward transport current (−8.28 nA ± 0.6). This membrane conductance exhibited a lower amplitude and inactivated rapidly (Figures 1B,C, left). The OCA-induced transient inward current was also elicited when OCA was perfused prior to DA exposure (data not shown).

Next, we set out to understand whether the OCA current was mediated by endogenous targets of OCA. One of the main targets of OCA, the G-Protein Coupled Receptor TGR5, is shown to be only minimally expressed in *Xenopus laevis* oocytes as reported in the platform Xenbase (Yanai et al., 2011; Session et al., 2016; James-Zorn et al., 2018). As shown in Figure 1B, no response from the perfusion of OCA was obtained in control oocytes (i.e., not expressing any heterologous protein). Thus, an activation of endogenous targets seems unlikely. As previously reported in literature, oocytes can express functional human TGR5 (hTGR5) (Lieu et al., 2014) as demonstrated by modulation of various membrane proteins upon TGR5 activation. We expressed hTGR5 in oocytes and measured OCA-induced currents. No currents were observed in hTGR5 expressing oocytes upon OCA perfusion (Figure 1B, right and Figure 1D). Next, we tested oocytes co-expressing mDAT and hTGR5. The currents recorded from these cells in response to DA (−12.88 nA ± 0.9) and OCA (−9.37 nA ± 0.82) alone (Figure 1B, center and Figure 1C, right) were comparable to the ones recorded in oocytes expressing mDAT alone (Figure 1D). This data suggests that the inward current induced by OCA is directly mediated by mDAT.

Dose-response experiments determined that 10 µM OCA was a saturating concentration. The EC50 was not calculated for this interaction because the current amplitude was consistent in response to over an eight order of magnitude range of OCA concentrations, as low as 1 pM and as high as 100 µM OCA (Figure 1E). The concentration of 10 µM was chosen throughout this study because it approximates the concentration of OCA in the plasma after oral administration and is sufficient to activate physiological targets of OCA *in vitro* (Roda et al., 2014).

To better characterize the OCA-induced current, we performed a voltage step protocol and obtained I/V curves for the transporter with DA and OCA (Figure 1F). As expected, DA-induced currents increased at negative potential without reverting. At positive voltages, the I/V curve for DA shows the distinctive inverted U shape, previously reported by Sonders and coworkers (Sonders et al., 1997), which is a hallmark of blockage of the tonic Na^+^-leak current. OCA displays a similar curve shape. However, it shows an increase in conductance at negative potentials and a lack of inversion at positive potentials (Figure 1F). These characteristics point towards OCA opening a conductance in mDAT.

### OCA Modulates the DAT Li^+^-Leak Current

**FIGURE 2:**
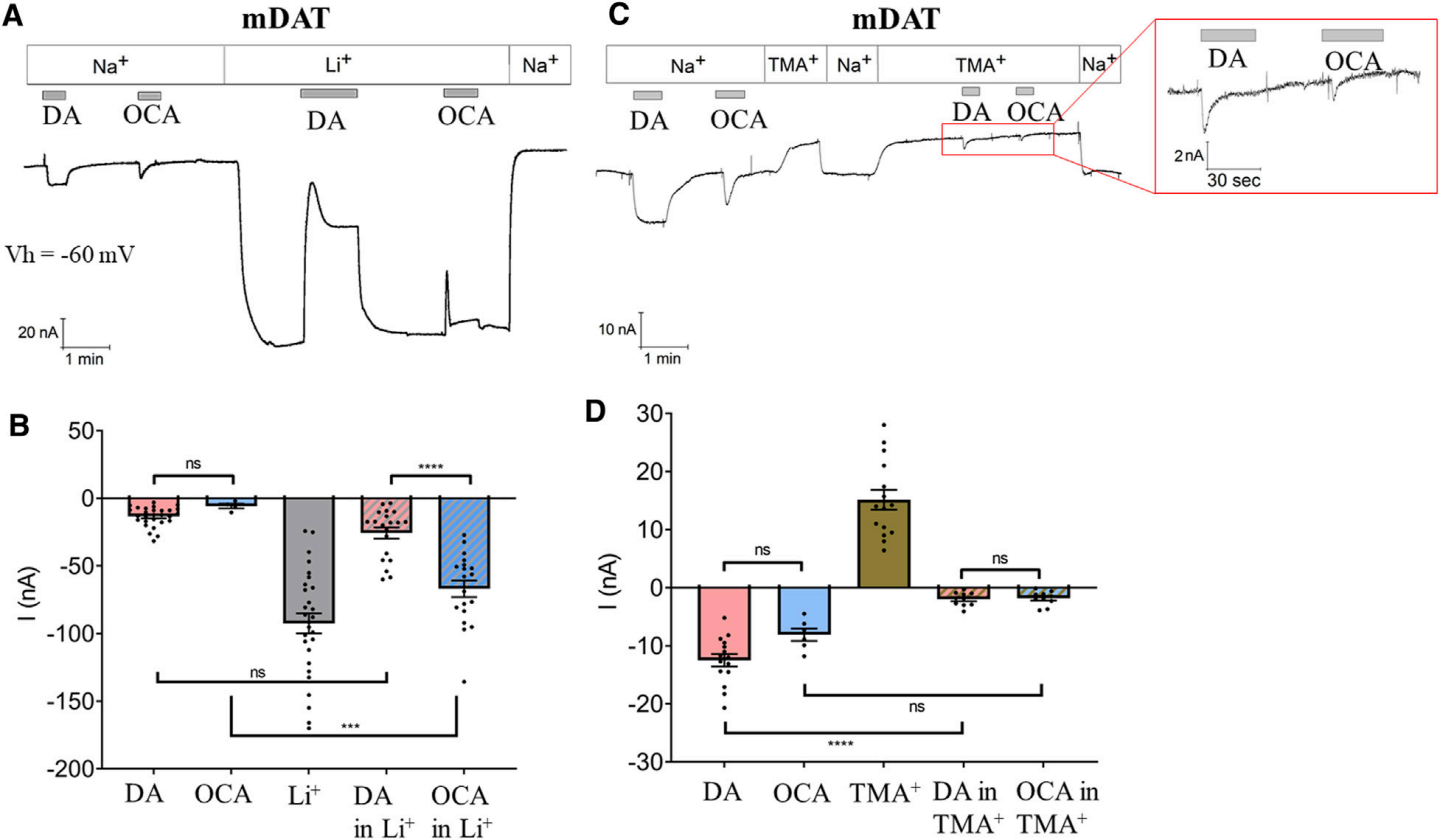
OCA modulates the mDAT-mediated leak current. **(A)** Representative current traces recorded with TEVC (Vh = −60 mV) in oocytes expressing mDAT perfused with 30 µM DA or 10 µM OCA in ND98 or Li98 buffer. **(B)** Mean of maximal currents under conditions shown in A (I nA ± SE of 6–28 oocytes, 4 batches. One-way ANOVA F(4,94) = 42.29, *p* < 0.0001 followed by Bonferroni’s multiple comparison test (DA vs OCA *p* > 0.999, DA vs DA in Li^+^ *p* > 0.999, OCA vs OCA in Li^+^ *p* = 0.0004, DA in Li^+^ vs OCA in Li^+^ *p* < 0.0001). **(C)** Representative trace from oocytes expressing mDAT perfused with 30 µM DA or 10 µM OCA in ND98 or TMA98 buffer. **(D)** Mean of maximal currents under conditions shown in C (I nA ± SE of 6–15 oocytes, 3 batches. One-way ANOVA F(4,51) = 86.94, *p* < 0.0001 followed by Bonferroni’s multiple comparison test (DA vs OCA *p* = 0.359, DA vs DA in TMA^+^ *p* < 0.0001, OCA vs OCA in TMA^+^ *p* = 0.059, DA in TMA^+^ vs OCA in TMA^+^ *p* > 0.999).

Several members of the solute carrier 6 (SLC6) family of transporters exhibit basal leak currents (Lester et al., 1994; Bossi et al., 1999; Andrini et al., 2008) that are augmented in the presence of Li^+^ ions. The Li^+^-leak current can be utilized to highlight the effect of molecules that interact with transporters and modify their electrical activity in the absence of Na^+^. DAT shows a significant Li^+^-leak current that is partially blocked upon DA perfusion (Giros et al., 1992; Sonders et al., 1997). Therefore, we used Li^+^ to investigate whether OCA, similarly to DA, binds mDAT in the absence of Na^+^. Oocytes were perfused first either with DA or OCA in Na^+^ bathing buffer (Figures 2A,B). Switching to Li^+^ bathing buffer induced a large inward current (−92.41 nA ± 7.45) (Figures 2A,B). As expected, the addition of DA to the Li^+^ buffer partially blocked the Li^+^-leak current (Figures 2A,B). This inhibition occurred in two phases; a rapid transient component (current at the peak: −25.72 nA ± 4.07) followed by a steady-state condition (−38.97 nA ± 5.11). After DA removal, the Li^+^-leak current returned to initial values (Figure 2A). Interestingly, perfusion of OCA also partially blocked the Li^+^-leak current (Figures 2A,B). As with DA, this inhibition displayed two phases; a transient rapid component (current at the peak: −66.86 nA ± 6.13) and a steady-state component (−82.42 nA ± 7.69). Inhibition of the Li^+^-leak current is a strong indication of direct binding of OCA to the transporter in the absence of Na^+^.

### mDAT-Mediated OCA Current Is Na^+^ Dependent

In addition to a coupled mechanism, Na^+^ also permeates through DAT in the absence of DA. This generates a leak current that can be uncovered when Na^+^ is substituted by non-permeant cations such as choline or TMA^+^ (Sonders et al., 1997). To better understand the effect of OCA on membrane conductance and the relevance of Na^+^ in the OCA-induced inward current, experiments were repeated with TMA^+^ as the cation substituting for Na^+^ in the bathing buffer. TMA^+^ blocked the Na^+^-leak current (15.12 nA ± 1.71) (Figures 2C,D). As expected, in the presence of TMA^+^, DA elicited only a fast-transient inward current (−1.98 nA ± 0.37), confirming that Na^+^ is necessary for mDAT-mediated DA currents. OCA behaved similarly to DA: the inward current was still present but greatly reduced in amplitude (−1.83 nA ± 0.38) (Figures 2C,D). The substitution of TMA^+^ for Na^+^ greatly affected the amplitude of the OCA-induced currents, pointing to a major role for the permeation of Na^+^ ions in causing the OCA-induced currents recorded in physiological solution.

### OCA-Induced Current Is Not Due to an Increase in Intracellular (IC) Calcium

**FIGURE 3:**
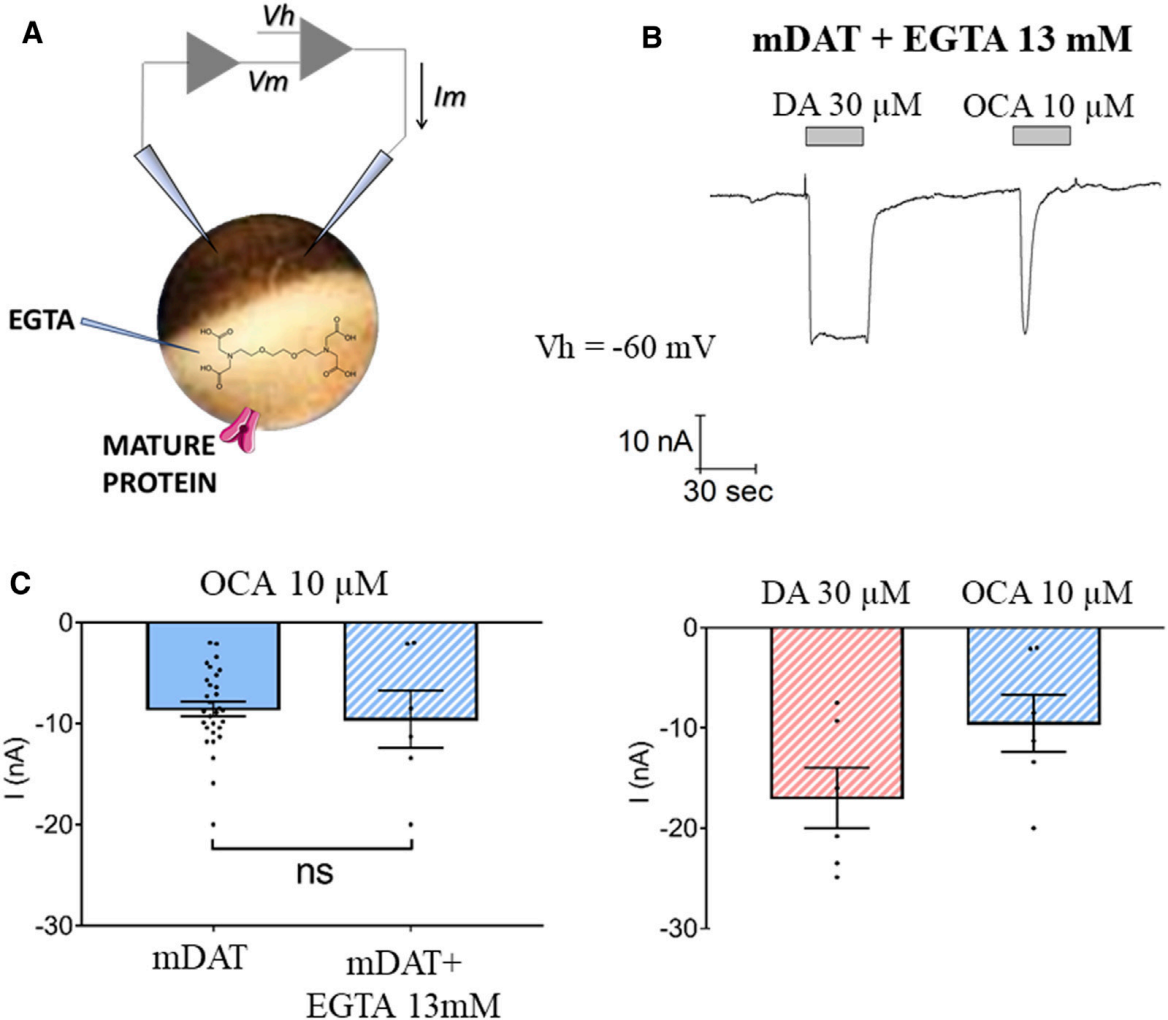
Intracellular calcium does not regulate OCA-induced currents. **(A)** Schematic representation of the EGTA injection technique. **(B)** Representative trace of current recorded by TEVC (Vh = −60 mV) in oocytes expressing mDAT and injected with 13 mM EGTA in intracellular solution 30 min before exposure to 30 µM DA or 10 µM OCA in ND98 buffer **(top)** and the mean of maximal transport-associated and OCA-induced currents **(bottom)** (I nA ± SE of 6–7 oocytes, 2 batches). **(C)** Mean of maximal OCA-induced currents in mDAT oocytes with or without injection of EGTA. Two-tailed Student’s *t*-test, *p* = 0.476.

OCA has been shown to induce IC Ca^2+^ fluctuations (Hao et al., 2017). Thus, it is possible that the OCA-induced inward current could be generated by the activation of chloride conductance due to an increase in IC Ca^2+^ concentrations. To investigate this possibility, experiments were conducted in oocytes expressing mDAT and injected with the Ca^2+^-chelating agent EGTA (Figure 3A). The presence of EGTA did not alter mDAT-mediated currents elicited by DA or OCA (Compare Figure 3B with Figures 1B,C). Maximal OCA-induced inward currents were also not altered by the presence or absence of EGTA (Figure 3C). Together, these data strongly suggest that IC Ca^2+^ does not regulate OCA-induced currents and further suggest that effects of OCA are mediated by direct interaction with mDAT.

### OCA Does Not Alter Either DAT-Mediated DA Currents or DA Affinity

**FIGURE 4:**
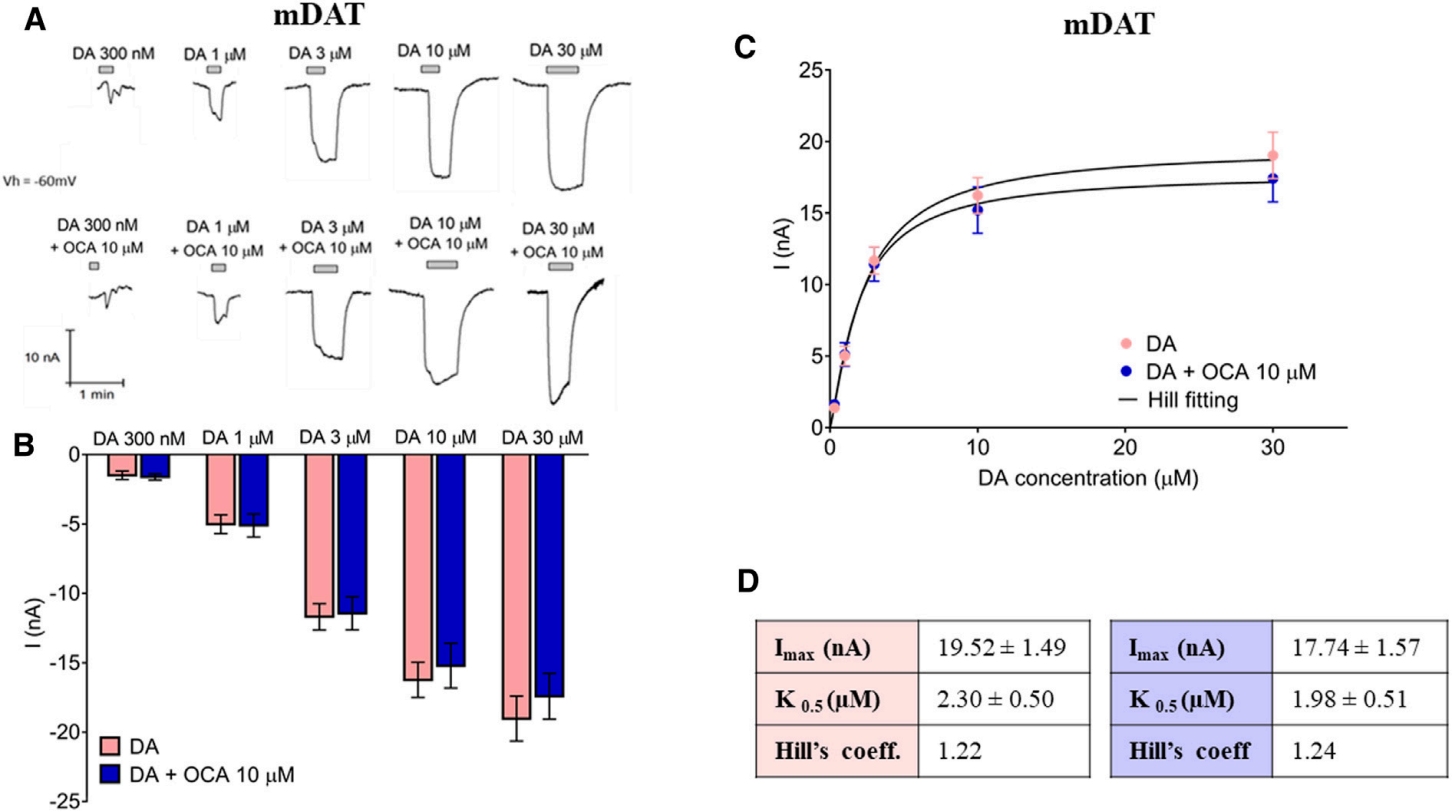
OCA does not alter the DA transport associated current. **(A)** Representative traces of the current recorded at increasing concentrations of DA in the absence or presence of 10 µM OCA. **(B)** Mean currents recorded from the traces in **(A)**. Two-way ANOVA, Treatment F(1,142) = 0.51, *p* = 0.476 – DA concentration F(4,142) = 77.76, *p* < 0.0001 – Interaction F(4,142) = 0.24, *p* = 0.915. **(C)** Data from **(A)** were fitted to a Hill equation. **(D)** Imax, K_0.5_, and Hill coefficient obtained from fitting the data represented in **(B)** to the Hill equation.

Data thus far indicate that OCA interacts directly with mDAT in the absence of DA. We next investigated whether OCA regulates DAT-mediated DA-induced currents. Currents generated from increasing concentrations of DA were unaltered by the presence of 10 µM OCA even at low DA concentration (Figures 4A,B). Mean of maximal currents were fitted to a Hill equation (Figure 4C) and the results reported in Figure 4D. These data indicated that the presence of OCA does not significantly affect either the affinity or the maximal transport currents.

### Lithocholic Acid Induces DAT-Mediated Currents

**FIGURE 5:**
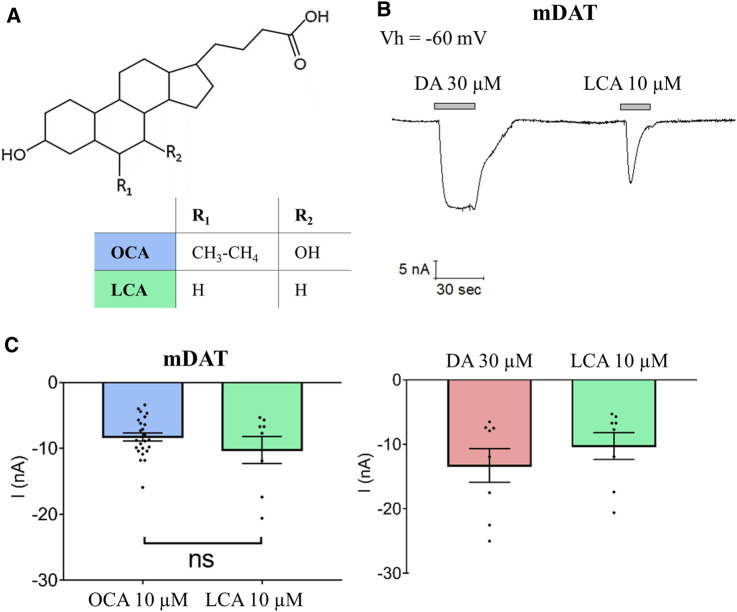
LCA induces a current that parallels the OCA-induced current. **(A)** Structure of OCA and LCA **(B)** Representative trace of current recorded with TEVC (Vh = −60 mV) in oocytes expressing mDAT and perfused with 30 µM DA or 10 µM LCA in ND98 buffer **(top)**. Mean of maximal dopamine transport-associated and LCA-induced currents (I nA ± SE of 8 oocytes, 3 batches) **(bottom)**. **(C)** Mean of maximal currents elicited by OCA or LCA in mDAT-expressing oocytes (*n* = 8; *p* = 0.22 by Student’s *t*-test).

To determine whether the OCA-induced mDAT-mediated current is specific to OCA, or is a common phenotype induced by bile acids, we investigated the effect of the natural bile acid, lithocholic acid (LCA). OCA and LCA share the same sterol-based structure with different R groups at positions 5 and 6 of the B ring (Figure 5A). Specifically, ethyl and hydroxyl groups present in OCA are substituted by hydrogen in LCA. Similar to OCA, perfusion of 10 µM LCA onto mDAT-expressing oocytes induced a transient inward current (−10.25 nA ± 2.06) (Figures 5B,C). Direct comparison of OCA and LCA revealed no significant differences in their ability to induce DAT-mediated currents (Figure 5C). These data suggest that the ability of OCA to promote DAT-mediated currents is shared by another BA.

### The Binding Pose of OCA(n) Suggests That It Binds to DAT in a Non-Competitive Fashion, That Allows Simultaneous Binding of DA

**FIGURE 6:**
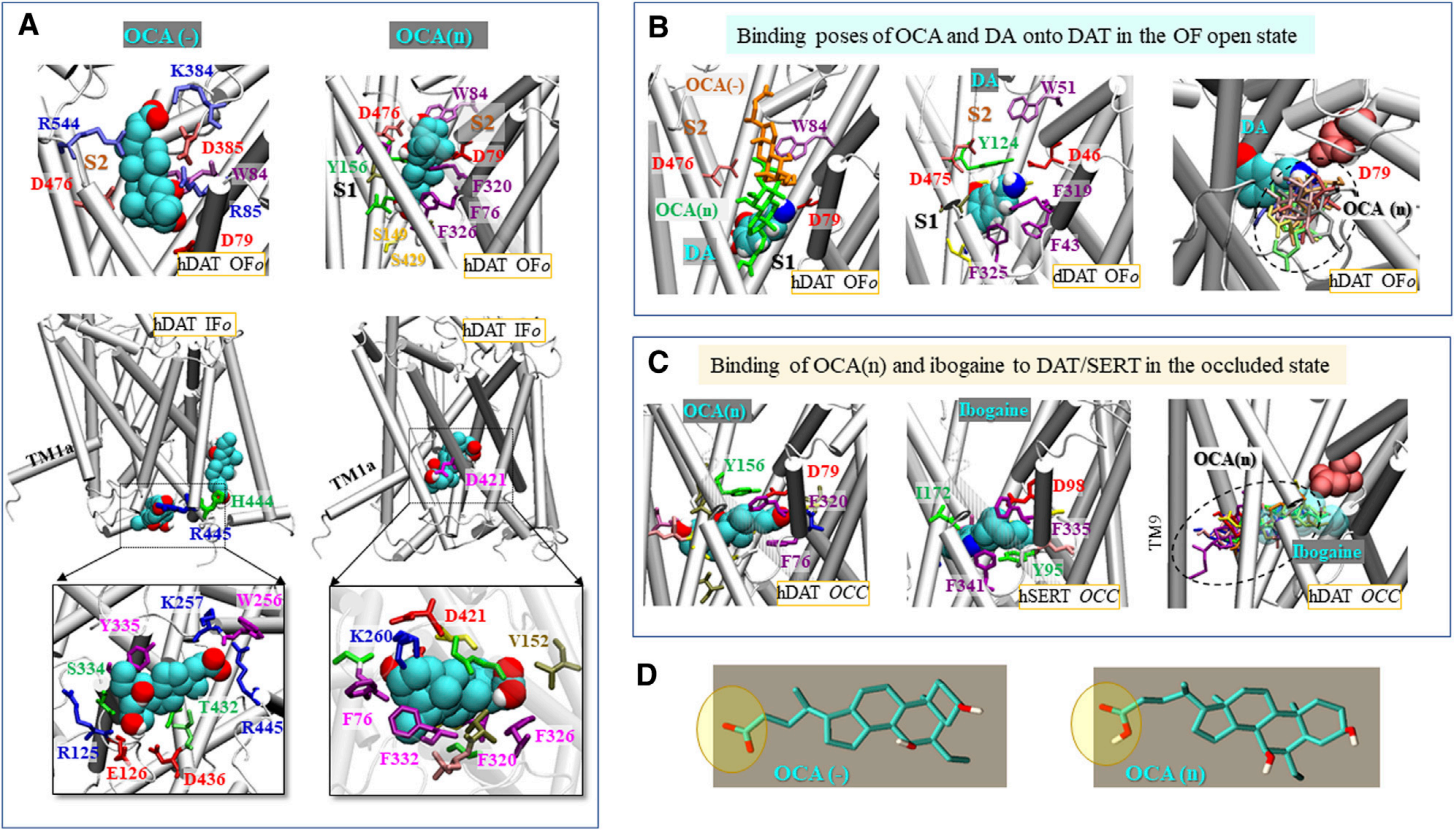
OCA binding to DAT depends on its protonation state and on DAT conformation. **(A)** Binding of negatively charged **(*left*)** and neutral **(*right*)** OCA (cyan) to hDAT in the OF*o* **(*top two panels*)** and IF*o* **(*middle and bottom panels*)** states. The binding energies to OF*o* hDAT are −6.1 kcal/mol (OCA(−)) and −9.9 kcal/mol (OCA(n)); and those to the IF*o* hDAT are −7.5 kcal/mol (OCA(−)) and −8.5 kcal/mol (OCA(n)). Interacting residues making atom-atom contacts closer than 4 Å with OCA are shown in panels **(A–C)**. **(B)** Comparison with the known DA-binding pose. Alignment of the top binding poses of OCA(n) (*green sticks*), OCA(−) (*orange sticks*) **(*left panel*)** and the resolved DA (van der Waals (vdW) format) bound dDAT (PDB: 4XP1) **(*middle panel*)**, and detailed view of DA-binding site and comparison of the non-overlapping spaces occupied by OCA (multiple binding poses) and DA **(*right panel*)**. **(C)** Top 1 binding pose of OCA(n) (vdW format; −10.2 kcal/mol) to hDAT occluded (OCC) conformer **(*left panel*)**, the resolved ibogaine-bound to hSERT in the OCC state (PDB:6DZV) **(*middle panel*)** and alignment, onto hDAT, of top 10 binding poses of OCA(n) (*sticks in different colors*; average = −9.1 ± 1.0 kcal/mol) and ibogaine bound to SERT (transparent vDW) **(*right panel*)**. OCA, DA, and ibogaine are shown in vdW format in **(A–C)**, with *cyan, red, blue* and *white spheres* representing the carbon, oxygen, nitrogen and hydrogen atoms, respectively. **(D)** Molecular structures of OCA in two protonation states.

To identify potential sites for binding of OCA onto mDAT and hDAT, docking simulations were performed, using AutoDock4 (Morris et al., 2009) and AutoDock Vina (Trott and Olson, 2010). To account for alternative protonated and deprotonated states of OCA, both the neutral (OCA(n)) and negatively-charged (OCA(−)) forms (Figure 6D) were used in the simulations. Computations were performed for the outward-facing *open* (OF*o*), *occluded* (OCC), and inward-facing *open* (IF*o*) states of both mDAT and hDAT. The computations revealed that OCA selected similar binding poses, with comparable binding affinities, for either transporter when the same conformational state was targeted regardless of the species. Therefore, representative results for hDAT are presented unless otherwise stated.

Figure 6A illustrates the top-ranking distinct binding poses and sites of both OCA forms, observed in docking simulations, onto hDAT in the OF*o* and IF*o* states in the absence of DA. Panel B shows the comparison to the DA-bound structures resolved for dDAT (Wang et al., 2015) in the presence of OCA, and panel C to ibogaine-bound hSERT (Coleman et al., 2019). Notably, in the case of the OF*o* conformer, the most favorable binding sites for both the neutral and negatively charged forms of OCA are within the extracellular (EC) vestibule but the exact locations are determined by the protonation state of the ligand. In the absence of DA, OCA(−) occupies the S2 site proposed to allosterically modulate transport (Cheng and Bahar, 2019), whereas OCA(n) binds an extended region spanning between the primary (S1) and secondary (S2) sites (Figure 6A, top panels). The binding pose of OCA(−) thus differs from that of the DA bound to OF*o* DAT (Figure 6B) where the residue equivalent to hDAT D79 (D46 in dDAT) plays a major role in coordinating the binding of the amine group through a salt bridge formation (Penmatsa et al., 2013; Wang et al., 2015; Cheng and Bahar, 2019). Notably, when DA is present, the top binding poses of OCA do not block the binding of substrate DA (Figure 6B, left panel), such that DA is able to bind in the proximity of OCA. This is consistent with the non-competitive binding of OCA revealed in TEVC experiments (Figure 4).

For the IF*o* conformer, no high affinity binding is observed to the EC vestibule. For OCA(n), the top binding site is in proximity to D421 within the IC vestibule (Figure 6A, bottom right panel). OCA(−) preferentially binds near the IC entrance, in the proximity to R445, and no high affinity binding is observed within the vestibule. Notably, docking simulations also revealed binding instances within the transmembrane region, including the site known to be occupied by cholesterol near TM1a (Penmatsa et al., 2013; Cheng and Bahar, 2019), or a site close to residues R443/H444 (Figure 6A, middle left panel) reported previously as a PIP_2_ binding site (Belovich et al., 2019). However, these sites could potentially be obstructed by lipid molecules, which are not included in docking simulations, and hence will not be further elaborated.

Unexpectedly, in the case of the *occluded* DAT conformer (Figure 6C), both charged and neutral OCAs (OCA(n/−)) are predicted to bind to the S1 site, with almost identical affinity, and the binding pose closely resembles that resolved for ibogaine-bound hSERT. Of note, the binding pocket in the occluded state may be much larger than anticipated, with possible involvement of TM9 (Figure 6C, right panel).

Taken together, these docking simulations suggest that the binding sites and affinities depend on the net charge carried by OCA and on the conformational state of the transporter. In the OF*o* state, the binding site for OCA(n) closely neighbors, but does not overlap with, the S1 site resolved for DA (Wang et al., 2015); whereas, in the absence of DA, the binding site for OCA(−) overlaps with the broadly-defined S2 site (Figure 6A). In the IF*o* state, on the other hand, OCA binds near D421 or R445, depending on its protonation state. Notably, in the occluded state of the transporter, OCA appears to select a binding site similar to that resolved for ibogaine-bound to hSERT (Coleman et al., 2019) regardless of its charge. However, caution is needed when interpreting docking-predicted binding sites, especially in the occluded state. The S1 site suggested in the *occluded* DAT conformer may not be accessible to OCA(−) in practice, even though it forms the pocket with the highest affinity, due to the fact that OCA(−) does not bind to the S1 site in the OF*o* conformer.

## Discussion

The comprehensive role of BAs in brain physiology is still unclear, but evidence of their involvement in various brain functions is continually increasing. BA concentrations in the brain strongly correlate with their plasma concentrations (Mano et al., 2004; Higashi et al., 2017). OCA, the main BA considered in this study, reaches a plasma concentration around 15 µM following intraduodenal administration in rats (Roda et al., 2014), comparable to the 10 μM concentrations used in bath applications for the TEVC studies reported here. Twenty BAs have been detected in brain lysates and most are present in forms loosely bound to proteins (Higashi et al., 2017; Kiriyama and Nochi, 2019). In contrast, cholesterol, which is highly lipophilic, is only soluble and present in plasma when tightly bound to plasma proteins (Cortes et al., 2014). As a consequence, regulation of DAT via cholesterol occurs upon intercalation into the plasma membrane (Hong and Amara, 2010; Zeppelin et al., 2018). However, the increased charge present on BAs likely precludes this mechanism for regulation of MATs via these molecules. Although our docking simulations revealed, among others, binding instances of OCA to the site known to be occupied by cholesterol (Penmatsa et al., 2013; Cheng and Bahar, 2019), this site is likely obstructed by higher affinity lipid molecules, which are not included in docking simulations, and hence were not further elaborated.

These current findings highlight previously undocumented interactions and impact of BAs on DAT function. Specifically, we show that this interaction promotes a current that is transient in nature and is Na^+^-dependent. OCA is capable of inhibiting, as observed for DA (Sonders et al., 1997), a DAT-mediated Li^+^-leak current suggesting that BAs can interact with DAT even in the absence of Na^+^. Furthermore, the OCA-induced current does not depend upon changes in intracellular calcium levels. In our interpretation, the presence of a DAT-mediated OCA current suggests that OCA could induce an occluded conformation of the transporter. This conformational state could transition to an outward facing conformer upon binding to substrate, as suggested by the competition experiments.

As per the nature of the current itself, the data obtained strongly suggest that the current generated by OCA is a DAT-mediated, Na^+^-dependent leak current. In previous *in silico* studies of DA-free DAT conformers (Cheng et al., 2018; Cheng and Bahar, 2019), the EC- and IC-exposed helices were not as tightly packed as in the *occluded* DA-bound form. These conformers occasionally gave rise to simultaneous opening of both the EC and IC gates such that formation of an intermittent water channel was detected. Notably, the sodium permeation pathway (Aguilar et al., 2021) coincides with that of water channeling (Cheng et al., 2018; Cheng and Bahar, 2019). This path, observed *in silico*, may also be associated with DAT-mediated ion fluxes or leak currents (Ingram et al., 2002; Erreger et al., 2008). Furthermore, Na^+^ substitution with TMA^+^ strongly reduces the ability of OCA to induce this current. The blocking of the Li^+^-leak current could be caused by binding of OCA, in the charged state, to the S2 substrate site, and its deeper insertion and translocation after protonation that may induce intramolecular rearrangements similar to DA. Differences were observed between the effects of OCA and DA on DAT conductance in terms of steady-state currents. This may be due to the fact that the release of DA and simultaneously-bound Na^+^ restores DAT into a transporter mode (Borre et al., 2014), whereas the non-substrate OCA will not facilitate full procession to this state resulting in a current that is transient. Considering that Li^+^ leakage in DAT is dependent on the Na2 site rather than the Na1 site (Borre et al., 2014), another possibility is that D421 in hDAT coordinates the binding of the Na^+^ ion to the Na2 site (Borre et al., 2014; Cheng and Bahar, 2015). Notably, the present docking simulations also indicate that D421 may contribute the binding of OCA(n) (Figure 6A) in the IF*o* state. The direct binding of OCA to D421 may also potentially block Li^+^ permeation. However, we cannot rule out the possibility that inhibition of the Li^+^ current by OCA may reflect a shift of conformational equilibrium between different functional states along the transport cycle, as proposed for DA (Borre et al., 2014).

The docking simulations also provide important insights into the potential functional relevance of the OCA-DAT interaction. Surprisingly, these results suggest that OCA binds a similar site to that resolved for ibogaine-bound hSERT (Coleman et al., 2019) in the *occluded* state (Figure 6C). Ibogaine is a non-competitive inhibitor for both DAT and SERT and has been proposed to stabilize the transporters in the IF conformation (Jacobs et al., 2007; Bulling et al., 2012). It is important to consider that compounds that bind to the S1 or S2 site (or at both sites) would induce an inhibition of substrate transport. However, we observed that OCA binds in a non-competitive way with respect to DA (Figure 4). One possibility is that the predominant form of OCA present in experiments carried a negative-charge and therefore did not bind the S1 site as modeled for OCA(−) in the OF*o* state (Figure 6, panel A). The negatively-charged OCA, in fact, binds only superficially near the entrance to the EC vestibule of DAT and could only exert a limited effect on DA transport. Further studies on the residues involved in the binding will aid in clarification of this point.

Of note, our model points to a separate site, of equal potential functional impact, for OCA on DAT; residues R445 and D436 (Figure 6A) which are involved in the formation of salt bridges in the IF conformer. In the DAT transport cycle, the binding of DA to S2 and S1 promotes the closure of the EC gate upon stabilization in the site S1. It then cooperatively restores the compact association of the EC vestibule while translocating to the IC vestibule (Cheng and Bahar, 2015). The IC vestibule opens to the cytoplasm in the IF conformation only after compaction/closure of the EC-exposed region (Cheng and Bahar, 2015). The binding of OCA may stabilize the IF*o* state by preventing the re-formation of the IF gating salt bridges, R60-D436 and R445-E428, that are required for DA transport (Cheng and Bahar, 2019). Recently, molecular modeling found that the infantile Parkinsonism-Dystonia associated substitution, R445C in hDAT, disrupted a phylogenetically conserved intracellular network of interactions and promoted a channel-like intermediate of hDAT. These rearrangements lead to the permeation of Na^+^ from both the EC and IC solutions (Aguilar et al., 2021). Interestingly, docking simulations suggested that OCA could also bind near H444/R445 to stabilize the IF state or act as an anion-lipid (Figure 6A for OCA(−) in the IF*o* state). Further experiments will be performed to confirm the importance of these predicted docking sites for the interaction of OCA and DAT.

Lastly, the findings reported in Figure 5 confirm that binding to DAT is not unique to OCA, but takes place also upon application of LCA, a natural bile acid. This observation is important as this class of molecules have not been previously reported to interact with MATs. Thus, BAs in general hold potential as novel pharmacological tools that could potentially impact important MAT functions, such as the channel-like activity of DAT.

## Data Availability

The datasets presented in this study can be found in online repositories. The names of the repository/repositories and accession number(s) can be found in the article.
